# Associations Between Match‐Play Characteristics and Environmental Temperatures in 4 Professional Football Leagues

**DOI:** 10.1002/ejsc.12256

**Published:** 2025-02-15

**Authors:** Edgar Schwarz, Rob Duffield, Andrew Roman Novak, Dennis Alan Compton, Tim Meyer

**Affiliations:** ^1^ Institute of Sports and Preventive Medicine Saarland University Saarbrücken Germany; ^2^ School of Sport, Exercise and Rehabilitation, Faculty of Health University of Technology Sydney Ultimo NSW Australia

**Keywords:** heat, optical tracking, performance analysis, team sport, temperature, thermoregulation, wet bulb globe temperature

## Abstract

This study investigated the association between environmental temperature and match‐play characteristics (shooting, passing, dribbling and defending) in four professional football leagues. Twenty‐seven performance indicators (PI's) were collated from 1585 matches from the German Bundesliga 1 and 2, Spanish La Liga and Australian A‐League. Environmental data were obtained for dry‐bulb temperature (T) and wet‐bulb globe temperature (WBGT) retrospectively from public sources. For each league, linear regressions were used to determine relationships between PI's and T and WBGT and linear mixed models were used to determine those associations across all four leagues. Individual leagues showed varying associations between a collection of PI's and environmental measures. When combining the four leagues' match data, 8 of the 17 investigated parameters were associated with T and WBGT (*p* < 0.002). Passes, especially short passes, were reduced in higher T (−2.3 [−3.1 to −1.5] and *p* < 0.001) and WBGT (−3.1 [−4.0 to −2.1] and *p* < 0.001), alongside an increase in the success rate of passes (0.06 [0.02–0.09] and *p* ≤ 0.001). The number of passes into the opponent's final third was reduced for both T (−0.18 [−0.25 to −0.05] and *p* = 0.001) and WBGT (−0.17 [−0.28 to−0.05] and *p* = 0.002), but the number of key passes leading to a shot or goal was not associated with T or WBGT (*p* ≥ 0.67). The number of touches, take‐ons and turnovers were reduced in higher T and WBGT (all *p* < 0.001). Accordingly, in higher heat stress, match actions, especially those performed at high volumes, are reduced. Therefore, teams should expect a possibly altered match play and may consider adapting tactical or heat‐mitigating strategies to counter these effects.

**Summary**

- In warmer environmental conditions, footballers perform less passes especially short passes and passes into the final third.
- In warmer environmental conditions, footballers perform less touches, touches in the final third and dribble take‐ons.
- In warmer environmental conditions, footballers' success rate of passes is higher and they perform less turnovers.

## 1 Introduction

Football matches played in hot conditions are of growing concern to football federations, clubs and players (Gouttebarge et al. 2023; Nassis et al. 2024). Although it is known that competing in hot environmental conditions can have implications for physical and cognitive performance and health, most football literature focusses on either thermoregulatory function or running demands (Draper et al. 2022; Maughan et al. 2010). However, research on the effects on football match play (i.e., number of match actions and success rates of technical parameters) remains lacking, especially given its importance to overall match performance (Broich et al. 2014; Chmura et al. 2021; Rampinini et al. 2009). Understanding match‐play characteristics in changing environmental temperatures can inform players and staff on expected technical and tactical performance changes and inform federations on the overall game style for viewing audiences.

During exercise, thermoregulatory function alleviates metabolic heat production. In environments where the air and mean radiant temperature are higher than an athlete's skin temperature, heat dissipation occurs predominantly through sweating (evaporative heat loss). However, in hot environments with high humidity or inadequate wind flow, evaporation of sweat is impaired and heat loss decreases (Bergeron et al. 2012). When heat gain exceeds loss, this can lead to increasing core temperatures linked to health adversities (Cramer and Jay 2016; Nybo, Rasmussen, and Sawka 2014). Nevertheless, among athletic populations high‐core temperatures are often sustained without negative health implications but are associated with performance changes (Racinais et al. 2023). In football, this typically translates into a reduced running performance (Draper et al. 2022; Schwarz et al. 2024). However, running is only one part of football, and whether higher environmental temperatures also result in deteriorations of other match actions (e.g., passes, touches and tackles) is yet to be investigated using a large multileague sample. The increased or earlier manifestation of fatigue due to hot conditions can result in alterations of cognitive and motor skill performance, such as the impairment of complex tasks namely decision‐making, working memory, recognition and problem‐solving (Schmit et al. 2017), which are slower or worse in hotter temperatures (Coehoorn et al. 2020; Syndicus et al. 2018). These effects on cognitive function, alongside observed alterations to pacing strategies (Racinais et al. 2015; Skein et al. 2012), may affect player's technical engagement, and thus affect team performance and match style. Hence, playing football in hot conditions could result in a lower playing quality, with players performing more errors (cognitive performance decrease) or reducing the quantity of match actions (team tactical or individual pacing approaches). On the other hand, playing football in colder conditions has not been found to affect performance (Carling, Dupont, and Le Gall 2011).

Only five observational studies have investigated changes to football match characteristics based on environmental conditions. These studies reported minor changes in performance indicators (PI's), such as passing (total passes and pass rates), shooting (goals scored and shots taken) and defending (number of duels) parameters. These investigations included environmental measurements of dry‐bulb temperature (T), relative humidity (RH), wet‐bulb globe temperature (WBGT) or universal thermal climate index (UTCI) (Chmura et al. 2021; Konefał et al. 2021; Nassis et al. 2015; Zhou et al. 2019). A higher pass rate was found during the 2014 FIFA World Cup in Brazil when environmental stress was ‘high’ compared to ‘low’ (Nassis et al. 2015). A similar small effect (*β* = 0.27) for increased pass rates in higher T was reported in Chinese first‐division football (Zhou et al. 2019). This was hypothesised to be linked to less opponent pressure caused by lower running distances in hotter conditions (Nassis et al. 2015; Zhou et al. 2019). However, other investigations have not observed this relationship (Chmura et al. 2021; Konefał et al. 2021), instead reporting fewer passes performed above 26° UTCI at the 2018 FIFA World Cup in Russia. Although these descriptions are useful, they investigated a small number of PI's during singular tournament settings, that is, the 64 matches of a single FIFA World Cup (Konefał et al. 2021; Nassis et al. 2015), or data from a single league (Chmura et al. 2021; Zhou et al. 2019). The different findings from these studies suggest that the influence of higher temperatures on match characteristics can vary between different locations and competition settings. Future research could investigate different settings and aim to include more factors (e.g., player acclimatisation and fitness, playing styles and competition structures) to identify potential reasons for such differences.

Many heat policies and guidelines exist to support players and staff in hot environments and limit possible health complications whilst maintaining performance (Gouttebarge et al. 2023; Nassis et al. 2024). Such heat policies commonly rely on WBGT as the underlying heat stress index and vary between federations. For example, the Fédération Internationale de Football Association (FIFA) suggests additional drinking breaks when WBGT exceeds 32°C, whereas Football Australia implements them at 26°C WBGT (Gouttebarge et al. 2023). Although widely used, WBGT has been criticised for its difficulty to measure and potentially underestimating the risk in humid conditions (Brocherie and Millet 2015). Although the purpose of these policies is to reduce heat‐illness risk, from a performance perspective, it is important to understand expected changes in match characteristics. Hence, this study aimed to investigate the relationship between environmental conditions and match PI's across four professional football leagues. We hypothesised that in higher WBGT or T, there would be significantly lower volumes of shooting, passing, dribbling and defending PI's, whilst performance quality metrics would be maintained.

## 2 Materials and Methods

This study observed 1585 matches (all played on natural grass) from four professional male football leagues across Germany, Spain and Australia. The German data were obtained over one season (2021–2022) of the first (BL1) and second (BL2) Bundesliga. The 306 matches, including match locations, kick‐off times and 143 match play PI's, were obtained through an open‐source website (FBREF) for each German league. For the Spanish league, match PI's from two seasons (2021–2023) of Spain's first division, La Liga (LL), were collected through the same website (FBREF), resulting in a total of 520 matches, with 67 PI's aligned to each match. Finally, data from Australia were provided for four seasons (2016–2020) of the Australian first division A‐League (AL), sharing match information for 453 matches and 109 PI's aligned with each match. All match‐play data were generated using ‘Opta’ (STATS PERFORM), an optical tracking service using a combination of human annotation, computer vision and artificial intelligence modelling. Prior to analysis, all individual player or team information was removed, and data were analysed as PI per match for both teams in that match. For example, in any match, if the two teams performed 500 and 400 passes, respectively, this was summed to a single metric (900 passes) to describe the passing volume for that match. After scanning all available PI's, a total of 17 PI's were deemed as relevant based on use in previous research and appropriateness to the topic, of which 15 were available across all four leagues, with two markers (passing distance and final third passes) not being available for the AL. Table 1 summarises all PI's in four categories: shooting, passing, dribbling and defending and gives detailed definitions of how each PI was defined. The ethical approval was granted by the Ethics Committee of the Faculty for Human and Business Sciences of Saarland University (Ref No.: 23‐14).

**TABLE 1:** Definitions of performance indicators (PI's).

Performance indicator	Definition
*Shooting*	
Goals	Number of goals scored
Shots	Number of shots taken
*Passing*	
Passes	Overall number of pass attempts (successful and unsuccessful)
Pass‐success rate	Percentage of pass attempts being successful
Short passes	Number of short passes (In BL1, BL2 and LL: shorter than 15 yards ≈ 13.7 m)
Long passes	Number of long passes (In BL1, BL2 and LL: longer than 30 yards ≈ 27.4 m)
Passing distance	Total distance (in metre) that completed passes have travelled in any direction
Key passes	Passes that directly lead to a shot
Final third passes	Passes that enter the third of the pitch closest to the opponent's goal
*Dribbling*	
Touches	Overall number of touches
Offensive touches	Number of touches in the offensive third
Take‐ons	Number of take‐ons
Take‐on success rate	Percentage of take‐ons being successful
*Defending*	
Turnovers	Total number of turnovers including unsuccessful pass attempts, unsuccessful take‐ons or other unsuccessful touches/mis‐controls resulting in the loss of possession
Tackles	Number of tackles
Fouls	Number of fouls committed
Cards	Number of yellow and red cards

Abbreviations: BL1 = Bundesliga 1; BL2 = Bundesliga 2 and LL = La Liga.

Environmental conditions in the form of T and WBGT were obtained retrospectively and aligned to each match based on the location and kick‐off times. T is the commonly reported ambient air temperature, whereas WBGT is an index developed to monitor human heat stress in hot thermal environments, adding the influence of RH, wind and solar radiation. The use, advantages and disadvantages of WBGT have been described extensively in previous research (Brocherie and Millet 2015; Budd 2008; Lemke and Kjellstrom 2012; Liljegren et al. 2008). We included both T and WBGT to refine the understanding of the differences between these measures and their associations with match‐play, to provide insights on the future use of these measures. For BL1, BL2 and LL matches, environmental data were obtained from Meteostat.net, an open‐source service providing hourly meteorological data for most geological locations. They collate environmental data from the four closest weather stations to the stadium whilst considering elevation differences and provide the following data: T, RH, dew point, wind speed, air pressure, total precipitation and the descriptive weather condition (i.e., cloudy and sunny), based on a weighted interpolation considering the distance and elevation difference of the weather stations to the stadium. Due to concerns about the accuracy of the wind speed inside the stadiums, airflow was set to 1 m/s to represent a minimum airflow generated by the movement of players, standardising this between matches. Further, the maximum solar radiation was estimated using the solar angle at the time and location of the match. With this data, WBGT can be estimated using a validated and reliable method (Patel, Mullen, and Santee 2013) developed by Liljegren et al. (2008) (Liljegren et al. 2008) and implemented into R‐code by Casanueva (2019). For AL matches, as some locations were not accessible through Meteostat.net (Meteostat.net), environmental conditions were obtained from the commercial provider UBIMET GmbH (UBIMET GmbH). Similarly to Metoestat.net, they collect data from multiple weather stations, radar and satellite data to estimate meteorological data at given ground locations and provide measurements of T, RH and WBGT. As an internal validation of the WBGT data based on Meteostat.net data, the WBGT estimation method used for the BL1, BL2 and LL data was performed with the AL data as well and then compared to the WBGT reported from the commercial provider UBIMET.com, resulting in a very good linear association (correlation coefficient *r* = 0.93). Although data from both providers aligns, there might be differences between the retrospectively fitted data and the actual conditions in the stadium, as wind speed and solar radiation might change locally (Chalmers, Anderson, and Jay 2020; Racinais et al. 2023). Fewer local variations exist for T and RH, which yield the most weight in the used WBGT estimations. Therefore, the standardised use of minimal airflow and maximum solar radiation may result in a small overestimation of WBGT. As this would be a systematic overestimation, this should influence the intercept but not the direction and slope of the association between WBGT and PI's. To display the exposure to hot environments in each of the presented leagues, matches were categorised into no (< 18.3°C WBGT), low (18.4°C–22.2°C WBGT), moderate (22.3°C–25.6°C WBGT), high (25.7°C–27.8°C WBGT) and extreme (> 27.9°C WBGT) risk of sustaining exertional heat illness (Roberts et al. 2023). For the analysis, T and WBGT were not categorised but instead treated as continuous variables.

For each individual league, linear regression models were used to investigate relationships between each PI and T or WBGT, respectively. To interpret the relationship, the estimate (Est) and 95% confidence interval (95% CI) and standardised estimate (*β*) and 95% confidence interval (*β*‐95% CI) explained variance (marginal *R* ^2^) and *p*‐value (*p*) were determined. Significant associations between PI's and environmental temperatures are presented as per 1°C T or WBGT increase. Depending on *β*, effects can be categorised into small (0.10–0.29), medium (0.30–0.49) and large (> 0.50), whereas *R* ^2^ can also be interpreted as small (0.02–0.08), medium (0.09–0.24) and large (> 0.25) (Cohen 1988). Additionally, to determine effects present across the four leagues, all matches were also combined into one large multileague dataset, including the 17 PI's and environmental conditions for all 1585 matches. Due to potential differences in data sources and PI definitions and environmental conditions throughout the leagues, we performed mixed linear models and included the leagues as a random effect, resulting in the following models:

- ***Model_0:*** *PI ∼ 1 + (1|league)*
- ***Model_T:*** *PI ∼ T + (1|league)*
- ***Model_WBGT:*** *PI ∼ WBGT + (1|league)*

Statistical significance was defined at a level of 5% or less for the *α*‐error (*p* < 0.05). As 17 different PI's were investigated, a Bonferroni correction was performed and the level of statistical significance adjusted to *p* < 0.00294. Analysis and visualisation were performed with R Studio 2023.12.1 using R version 4.3.2 (R Core Team 2023) stats (R Core Team 2023), lme4 (Bates et al. 2015), ggplot2 (Wickham 2016) and jtools (Long 2022) libraries. Results are first reported for each individual league and then for the combined data.

## 3 Results

Environmental conditions across all leagues ranged from −1.7°C to 37.1°C T and −2.0°C to 29.6°C WBGT, with the AL having the warmest average conditions (T: 21.0 ± 5.3°C and WBGT: 18.1 ± 4.3°C), followed by the Spanish LL (T: 18.8 ± 6.8°C and WBGT: 15.5 ± 5.6°C) and then the two German leagues (BL1: T: 11.2 ± 7.1°C and WBGT: 8.9 ± 6.1°C and BL2: T: 11.5 ± 7.3°C and WBGT: 9.8 ± 6.6°C). In the A‐League, 1% of matches were held at extreme, 1% were held at high, 16% were held at moderate and 34% were held at a low heat risk. In Bundesliga 1 and 2, no matches were held at extreme or high risk, 1% and 3% were held at moderate and 8% and 11% were held at a low‐heat risk. In La Liga, no matches were held at extreme, 1% were held at high, 11% were held at moderate and 22% were held at a low‐heat risk. Overall, 8 out of the 17 investigated PI's were associated with T and WBGT across three categories: passing, dribbling and defending (*p* < 0.002). Shooting was the only category in which no significant association existed for all leagues (*p* > 0.04). All detailed results for each individual league and the combined dataset are summarised in Tables 2, 3, 4, 5, and in the following sections, changes are presented as per 1°C T or WBGT increase.

**TABLE 2:** Association between shooting PI's and environmental conditions.

	Mean ± SD	Temperature				Wet‐bulb globe temperature			
		Estimate (95% CI)	*β* (95% CI)	*R* ^2^ (marg.)	*p*‐value	Estimate (95% CI)	*β* (95% CI)	*R* ^2^ (marg.)	*p*‐value
Goals									
AL	3.0 ± 1.8	0.03 (−0.002–0.06)	0.09 (−0.01–0.18)	0.01	0.07	0.04 (0.004–0.08)	0.1 (0.01–0.19)	0.01	0.03
BL1	3.1 ± 1.8	0.00 (−0.03–0.02)	−0.02 (−0.13–0.10)	0.00	0.77	0.00 (−0.03–0.03)	0.00 (−0.11–0.11)	0.00	1.0
BL2	2.9 ± 1.5	0.00 (−0.03–0.02)	−0.03 (−0.15–0.08)	0.00	0.6	−0.01 (−0.03–0.02)	−0.03 (−0.14–0.08)	0.00	0.55
LL	2.5 ± 1.7	0.00 (−0.02–0.02)	0.00 (−0.09–0.09)	0.00	1.0	0.00 (−0.03–0.02)	−0.01 (−0.14–0.08)	0.00	0.8
Combi	2.8 ± 1.7	0.00 (−0.01–0.01)	0.007 (−0.05–0.07)	0.00	0.79	0.003 (−0.01–0.02)	0.01 (−0.05–0.07)	0.00	0.69
Shots									
AL	26.8 ± 6.9	0.12 (−0.004–0.24)	0.09 (0.00–0.18)	0.01	0.06	0.08 (−0.07–0.23)	0.05 (−0.04–0.14)	0.00	0.29
BL1	25.6 ± 5.8	−0.07 (−0.16–0.02)	−0.08 (−0.20–0.03)	0.00	0.15	−0.06 (−0.17–0.04)	−0.07 (−0.18–0.04)	0.00	0.23
BL2	27.6 ± 5.5	−0.06 (−0.14–0.03)	−0.08 (−0.19–0.04)	0.00	0.18	−0.06 (−0.15–0.04)	−0.07 (−0.18–0.05)	0.00	0.24
LL	23.5 ± 5.2	0.00 (−0.06–0.06)	0.003 (−0.08–0.09)	0.00	0.93	−0.02 (−0.10–0.06)	−0.‐02 (−0.11–0.07)	0.00	0.64
Combi	29.0 ± 8.5	−0.007 (−0.05–0.04)	−0.01 (−0.07–0.05)	0.06	0.78	−0.02 (−0.08–0.03)	−0.03 (−0.09–0.03)	0.00	0.37

Abbreviations: AL = A‐League; BL1 = Bundesliga 1; BL2 = Bundesliga 2; Combi = combined dataset including data from all 4 leagues; Estimate (95% CI) = estimate and 95% confidence interval; LL = La Liga; Mean ± SD = mean and standard deviation; *p*‐value (*) = significant after Bonferroni correction; *R* ^2^ (marg.) = marginal *R* ^2^: explained variance of the fixed effects; *β* (95% CI) = standardised estimate and 95% confidence interval.

**TABLE 3:** Association between passing PI's and environmental conditions.

	Mean ± SD	Temperature				Wet‐bulb globe temperature			
		Estimate (95% CI)	*β* (95% CI)	*R* ^2^ (marg.)	*p*‐value	Estimate (95% CI)	*β* (95% CI)	*R* ^2^ (marg.)	*p*‐value
Passes									
AL	938.8 ± 118.0	−3.38 (−5.42–−1.35)	−0.15 (−0.24–−0.06)	0.02	0.002*	−5.81 (−8.32–−3.31)	−0.21 (−0.30–−0.12)	0.04	< 0.001*
BL1	867.7 ± 117.7	−2.35 (−4.13–−0.56)	−0.15 (−0.26–−0.04)	0.02	0.01	−3.00 (−5.05–−0.95)	−0.16 (−0.27–−0.05)	0.02	0.004
BL2	795.9 ± 81.8	−1.64 (−2.88–−0.39)	−0.15 (−0.26–−0.03)	0.02	0.01	−1.74 (−3.13–−0.35)	−0.14 (−0.25–−0.03)	0.02	0.01
LL	943.4 ± 104.2	−2.19 (−3.50–−0.89)	−0.14 (−0.23–−0.06)	0.02	< 0.001*	−2.94 (−4.51–−1.36)	−0.16 (−0.24–−0.07)	0.02	< 0.001*
Combi	898.8 ± 121.4	−2.27 (−3.05–1.46)	−0.17 (−0.22–−0.11)	0.02	< 0.001*	−3.05 (−3.97–−2.11)	−0.19 (−0.25–−0.13)	0.02	< 0.001*
Short passes									
AL	768.7 ± 120.3	−3.28 (−5.36–−1.20)	−0.14 (−0.24–−0.05)	0.02	0.002*	−5.88 (−8.44–−3.33)	−0.21 (−0.30–−0.12)	0.04	< 0.001*
BL1	379.8 ± 67.1	−2.0 (−3.04–−0.96)	−0.21 (−0.32–−0.10)	0.04	< 0.001*	−2.39 (−3.59–−1.19)	−0.22 (−0.33–−0.11)	0.04	< 0.001*
BL2	340.9 ± 46.0	−1.79 (−2.47–−1.11)	−0.28 (−0.39–−0.18)	0.08	< 0.001*	−1.99 (−2.74–−1.23)	−0.28 (−0.39–−0.17)	0.08	< 0.001*
LL	377.4 ± 70.8	−0.93 (−1.83–−0.04)	−0.09 (−0.18–0.00)	0.01	0.04	−1.24 (−2.31–−0.16)	−0.10 (−0.18–−0.01)	0.01	0.02
Combi	482.7 ± 200.0	−1.81 (−2.43–−1.81)	−0.17 (−0.23–−0.11)	0.00	< 0.001*	−2.47 (−3.20–−1.74)	−0.20 (−0.26–−0.14)	0.00	< 0.001*
Long passes									
AL	120.7 ± 182.4	−0.21 (−0.53–0.11)	−0.06 (−0.15–0.03)	0.00	0.19	−0.09 (−0.48–0.31)	−0.02 (−0.11–0.07)	0.00	0.67
BL1	159.3 ± 23.4	0.13 (−0.24–0.50)	0.04 (−0.07–0.15)	0.00	0.48	0.15 (−0.29–0.58)	0.04 (−0.07–0.15)	0.00	0.49
BL2	166.8 ± 20.4	0.34 (0.03–0.65)	0.12 (0.01–0.23)	0.01	0.03	0.40 (0.05–0.75)	0.13 (0.02–0.24)	0.01	0.02
LL	159.1 ± 22.4	−0.54 (−0.82–−0.26)	−0.16 (−0.25–−0.08)	0.03	< 0.001*	−0.77 (−1.11–−0.44)	−0.20 (−0.28–−0.11)	0.04	< 0.001*
Combi	149.7 ± 28.1	−0.12 (−0.28–0.03)	−0.05 (−0.10–0.01)	0.00	0.13	−0.14 (−0.32–0.05)	−0.04 (−0.10–0.02)	0.00	0.15
Pass‐success rate									
AL	76.33 ± 4.16	−0.02 (−0.08–0.06)	−0.02 (−0.11–0.07)	0.00	0.67	−0.07 (−0.15–0.02)	−0.07 (−0.16–0.02)	0.00	0.14
BL1	81.75 ± 3.77	0.05 (−0.01–0.11)	0.09 (−0.02–0.2)	0.01	0.11	0.06 (−0.01–0.13)	0.1 (−0.02–0.21)	0.01	0.09
BL2	79.68 ± 3.95	0.06 (0.00–0.12)	0.12 (0.01–0.23)	0.01	0.04	0.08 (0.01–0.15)	0.13 (0.02–0.24)	0.01	0.02
LL	77.83 ± 5.15	0.11 (0.04–0.17)	0.14 (0.06–0.23)	0.02	0.001*	0.13 (0.05–0.21)	0.15 (0.06–0.23)	0.02	0.001*
Combi	77.99 ± 4.62	0.06 (0.02–0.09)	0.10 (0.04–0.16)	0.01	0.001*	0.06 (0.02–0.10)	0.09 (0.03–0.15)	0.01	0.002*
Key passes									
AL	17.9 ± 5.4	0.08 (−0.02–0.17)	0.08 (−0.01–0.17)	0.00	0.10	0.05 (−0.07–0.16)	0.04 (−0.05–0.13)	0.00	0.41
BL1	18.9 ± 4.8	−0.02 (−0.10–0.05)	−0.04 (−0.15–0.08)	0.00	0.54	−0.02 (−0.10–0.07)	−0.02 (−0.14–0.09)	0.00	0.7
BL2	20.1 ± 4.5	0.01 (−0.06–0.08)	0.01 (−0.10–0.13)	0.00	0.80	0.01 (−0.07–0.08)	0.01 (−0.10–0.12)	0.00	0.86
LL	17.6 ± 4.6	−0.03 (−0.07–0.05)	−0.01 (−0.10–0.07)	0.00	0.35	−0.01 (−0.1–0.37)	−0.04 (−0.13–0.05)	0.00	0.74
Combi	18.4 ± 4.9	0.00 (−0.03–0.04)	0.01 (−0.05–0.06)	0.00	0.83	−0.01 (−0.05–0.03)	−0.01 (−0.07–0.04)	0.00	0.65
Passes into final third									
BL1	54.9 ± 10.6	−0.27 (−0.44–−0.11)	−0.18 (−0.29–−0.07)	0.03	0.001*	−0.3 (−0.49–−0.11)	−0.17 (−0.29–−0.06)	0.03	0.002*
BL2	54.2 ± 10.7	−0.20 (−0.36–−0.03)	−0.13 (−0.25–−0.02)	0.01	0.02	−0.20 (−0.38–−0.02)	−0.12 (−0.23–−0.01)	0.01	0.03
LL	57.2 ± 12.6	−0.08 (−0.24–0.08)	−0.04 (−0.13–−0.04)	0.00	0.35	−0.08 (−0.27–0.12)	−0.03 (−0.12–0.05)	0.00	0.43
Combi	55.8 ± 11.6	−0.18 (−0.25–−0.05)	−0.11 (−0.17–−0.04)	0.01	0.001*	−0.17 (−0.28–−0.05)	−0.10 (−0.16–−0.03)	0.01	0.002*
Pass distance									
BL1	12.5 ± 1.6	−7.25 (−32.99–18.48)	−0.03 (−0.14–0.08)	0.00	0.58	−10.63 (−40.35–19.10)	−0.04 (−0.15–0.07)	0.00	0.48
BL2	12.7 ± 1.5	10.3 (−12.97–33.56)	0.05 (−0.06–0.16)	0.00	0.38	14.89 (−11.03–40.81)	0.06 (−0.05–0.18)	0.00	0.26
LL	13.3 ± 1.8	−9.6 (−32.97–13.77)	−0.04 (−0.12–0.05)	0.00	0.42	−17.58 (−45.8–10.65)	−0.05 (−0.14–0.03)	0.00	0.22
Combi	12.1 ± 1.6	−1.96 (−15.09–11.25)	−0.01 (−0.08–0.06)	0.00	0.77	−3.90 (−19.24–11.44)	−0.02 (−0.08–0.05)	0.00	0.62

Abbreviations: AL = A‐League; BL1 = Bundesliga 1; BL2 = Bundesliga 2; Combi = combined dataset including data from all 4 leagues; Estimate (95% CI) = estimate and 95% confidence interval; LL = La Liga; Mean ± SD = mean and standard deviation; *p*‐value (*) = significant after Bonferroni correction; *R* ^2^ (marg.) = marginal *R* ^2^: explained variance of the fixed effects; *β* (95% CI) = standardised estimate and 95% confidence interval.

**TABLE 4:** Association between dribbling PI's and environmental conditions.

	Mean ± SD	Temperature				Wet‐bulb globe temperature			
		Estimate (95% CI)	*β* (95% CI)	*R* ^2^ (marg.)	*p*‐value	Estimate (95% CI)	*β* (95% CI)	*R* ^2^ (marg.)	*p*‐value
Touches									
AL	1304.0 ± 113.5	−4.34 (−6.28–−2.39)	−0.2 (−0.29–−0.11)	0.04	< 0.001*	−6.87 (−9.25–−4.49)	−0.26 (−0.35–−0.17)	0.06	< 0.001*
BL1	1276.5 ± 118.7	−3.21 (−5.06–−1.36)	−0.19 (−0.30–−0.08)	0.03	< 0.001*	−3.95 (−6.08–−1.82)	−0.20 (−0.32–−0.09)	0.04	< 0.001*
BL2	1243.8 ± 149.7	−3.18 (−4.39–−1.98)	−0.29 (−0.39–−0.18)	0.08	< 0.001*	−3.4 (−4.76–−2.06)	−0.27 (−0.38–−0.17)	0.17	< 0.001*
LL	1148.4 ± 102.5	−2.74 (−4.02–−1.46)	−0.18 (−0.27–−0.1)	0.03	< 0.001*	−3.65 (−5.18 ‐ ‐ 2.11)	−0.2 (−0.29–−0.12)	0.04	< 0.001*
Combi	1212.8 ± 126.2	−3.25 (−4.05–−2.44)	−0.23 (−0.29–−0.17)	0.04	< 0.001*	−4.22 (−5.15–−3.27)	−0.26 (−0.32–−0.20)	0.04	< 0.001*
Touches offensive									
AL	271 ± 48.7	−0.54 (−1.39–−0.31)	−0.06 (−0.15–0.03)	0.00	0.21	−1.33 (−2.38–−0.28)	−0.12 (−0.21–−0.02)	0.01	0.01
BL1	263.4 ± 38.8	−1.13 (−1.73–−0.52)	−0.21 (−0.32–−0.10)	0.04	< 0.001*	−1.33 (−2.03–−0.64)	−0.21 (−0.32–−0.10)	0.04	< 0.001*
BL2	266.2 ± 35.4	−1.44 (−1.95–−0.92)	−0.3 (−0.41–−0.19)	0.09	< 0.001*	−1.61 (−2.18–−1.03)	−0.30 (−0.41–−0.19)	0.09	< 0.001*
LL	277.9 ± 46.2	−0.68 (−1.26–−0.10)	−0.1 (−0.19–−0.01)	0.01	0.02	−0.84 (−1.54–−0.14)	−0.10 (−0.19–−0.02)	0.01	0.02
Combi	270.9 ± 44.0	−0.9 (−1.21–−0.56)	−0.16 (−0.22–−0.10)	0.02	< 0.001*	−1.20 (−1.57–−0.80)	−0.18 (−0.24–−0.13)	0.03	< 0.001*
Take‐ons									
AL	34.0 ± 8.7	−0.17 (−0.32–−0.01)	−0.1 (−0.19–−0.01)	0.01	0.03	−0.25 (−0.44–−0.06)	−0.12 (−0.21–−0.03)	0.01	0.01
BL1	32.9 ± 8.6	−0.1 (−0.25–0.04)	−0.08 (−0.19–0.03)	0.00	0.15	−0.12 (−0.29–0.04)	−0.08 (−0.19–0.03)	0.00	0.15
BL2	34.2 ± 8.4	−0.22 (−0.34–−0.10)	−0.2 (−0.31–−0.09)	0.04	< 0.001*	−0.22 (−0.36–−0.09)	−0.18 (−0.30–−0.07)	0.03	0.001*
LL	31.6 ± 7.9	−0.03 (−0.13–0.07)	−0.03 (−0.11–0.06)	0.00	0.57	−0.05 (−0.17–0.07)	−0.04 (−0.12–0.05)	0.00	0.38
Combi	32.3 ± 8.4	−0.11 (−0.17–−0.05)	−0.10 (−0.16–−0.05)	0.01	< 0.001*	−0.14 (−0.21–−0.06)	−0.11 (−0.17–−0.05)	0.01	< 0.001*
Take‐on rate									
AL	52.76 ± 9.64	0.33 (0.16–0.49)	0.18 (0.09–0.27)	0.03	< 0.001*	0.37 (0.16–0.58)	0.16 (0.07–0.25)	0.02	< 0.001*
BL1	54.35 ± 9.96	0.06 (−0.10–0.22)	0.04 (−0.07–0.16)	0.00	0.44	0.07 (−0.11–0.26)	0.05 (−0.07–0.16)	0.00	0.43
BL2	52.10 ± 9.79	0.01 (−0.14–0.16)	0.007 (−0.11–0.12)	0.00	0.90	0.04 (−0.13–0.21)	0.03 (−0.09–0.14)	0.00	0.63
LL	52.61 ± 12.65	−0.35 (−0.51–−0.20)	−0.19 (−0.28–−0.11)	0.03	< 0.001*	−0.46 (−0.65–−0.27)	−0.2 (−0.29–0.12)	0.04	< 0.001*
Combi	52.86 ± 10.90	−0.05 (−0.12–0.02)	−0.04 (−0.09–0.02)	0.00	0.18	−0.06 (−0.15–0.02)	−0.04 (−0.10–0.01)	0.00	0.14

Abbreviations: AL = A‐League; BL1 = Bundesliga 1; BL2 = Bundesliga 2; Combi = combined dataset including data from all 4 leagues; Estimate (95% CI) = estimate and 95% confidence interval; LL = La Liga; Mean ± SD = mean and standard deviation; *p*‐value (*) = significant after Bonferroni correction; *R* ^2^ (marg.) = marginal *R* ^2^: explained variance of the fixed effects; *β* (95% CI) = standardised estimate and 95% confidence interval.

**TABLE 5:** Association between defending PI's and environmental conditions.

	Mean ± SD	Temperature				Wet‐bulb globe temperature			
		Estimate (95% CI)	*β* (95% CI)	*R* ^2^ (marg.)	*p*‐value	Estimate (95% CI)	*β* (95% CI)	*R* ^2^ (marg.)	*p*‐value
Tackles									
AL	35.1 ± 8.0	−0.1 (−0.24–0.04)	−0.06 (−0.16–0.03)	0.00	0.17	−0.08 (−0.26–0.09)	−0.05 (−0.14–0.05)	0.00	0.34
BL1	32.2 ± 7.7	−0.06 (−0.18–0.06)	−0.06 (−0.17–0.06)	0.00	0.33	−0.07 (−0.21–0.07)	−0.06 (−0.17–0.05)	0.00	0.31
BL2	30.1 ± 6.8	−0.04 (−0.16–0.04)	−0.07 (−0.18–0.05)	0.00	0.42	−0.05 (−0.19–0.04)	−0.07 (−0.18–0.04)	0.00	0.36
LL	31.4 ± 7.3	0.02 (−0.07–0.12)	0.02 (−0.06–0.11)	0.00	0.60	0.02 (−0.09–0.13)	0.02 (−0.07–0.10)	0.00	0.69
Combi	32.2 ± 7.7	−0.03 (−0.08–0.03)	−0.03 (−0.09–0.03)	0.00	0.30	−0.03 (−0.10–0.03)	−0.03 (−0.09–0.03)	0.00	0.32
Fouls									
AL	27.6 ± 6.3	−0.15 (−0.26–−0.04)	−0.13 (−0.22–−0.04)	0.01	0.01	−0.11 (−0.25–0.02)	−0.08 (−0.17–0.01)	0.00	0.1
BL1	22.7 ± 5.4	0.04 (−0.05–0.12)	0.05 (−0.07–0.16)	0.00	0.41	0.04 (−0.06–0.14)	0.05 (−0.07–0.16)	0.00	0.42
BL2	22.1 ± 5.2	0.03 (−0.05–0.11)	0.04 (−0.07–0.15)	0.00	0.48	0.02 (−0.07–0.11)	0.03 (−0.08–0.14)	0.00	0.62
LL	26.5 ± 5.7	−0.02 (−0.09–0.06)	−0.02 (−0.11–0.07)	0.00	0.66	−0.01 (−0.09–0.08)	0.01 (−0.09–0.08)	0.00	0.86
Combi	25.2 ± 6.1	−0.02 (−0.06–0.03)	−0.02 (−0.08–0.04)	0.00	0.45	−0.00 (−0.05–0.04)	−0.00 (−0.06–0.06)	0.00	0.9
Cards									
AL	4.1 ± 1.9	0.00 (−0.04–0.03)	0.01 (−0.10–0.09)	0.00	0.88	0.02 (−0.02–0.06)	0.04 (−0.05–0.14)	0.00	0.35
BL1	3.5 ± 1.9	0.02 (−0.01–0.05)	0.06 (−0.05–0.18)	0.00	0.27	0.02 (−0.01–0.05)	0.07 (−0.05–0.18)	0.00	0.25
BL2	4.1 ± 2.0	0.02 (−0.01–0.05)	0.06 (−0.06–0.17)	0.00	0.32	0.02 (−0.02–0.05)	0.06 (−0.05–0.17)	0.00	0.29
LL	5.6 ± 2.8	0.01 (−0.03–0.04)	0.02 (−0.07–0.10)	0.00	0.71	0.02 (−0.02–0.06)	0.04 (−0.05–0.12)	0.00	0.84
Combi	4.5 ± 2.4	0.01 (−0.01 – 0.03)	0.03 (−0.03–0.09)	0.00	0.33	0.02 (−0.00–0.04)	0.05 (−0.01–0.11)	0.00	0.08
Turnovers									
AL	234.8 ± 27.8	−0.76 (−1.24–−0.28)	−0.14 (−0.24–−0.05)	0.02	0.002*	−0.96 (−1.56–−0.36)	−0.15 (−0.24–−0.06)	0.02	0.002*
BL1	281.8 ± 35.6	−1.11 (−1.66–−0.56)	−0.22 (−0.33–−0.11)	0.05	< 0.001*	−1.31 (−1.95–−0.68)	−0.23 (−0.34–−0.12)	0.05	< 0.001*
BL2	285.4 ± 36.8	−1.25 (−1.83–−0.74)	−0.26 (−0.36–−0.15)	0.06	< 0.001*	−1.43 (−2.08–−0.86)	−0.26 (−0.37–−0.15)	0.06	< 0.001*
LL	253.4 ± 39.8	−1.62 (−2.11–−1.14)	−0.28 (−0.36–−0.20)	0.08	< 0.001*	−2.00 (−2.58–−1.41)	−0.28 (−0.37–−0.20)	0.08	< 0.001*
Combi	259.7 ± 40.7	−1.27 (−1.53–−1.02)	−0.28 (−0.34–−0.22)	0.06	< 0.001*	−1.53 (−1.84–−1.23)	−0.29 (−0.35–−0.23)	0.07	< 0.001*

Abbreviations: AL = A‐League; BL1 = Bundesliga 1; BL2 = Bundesliga 2; Combi = combined dataset including data from all 4 leagues; Estimate (95% CI) = estimate and 95% confidence interval; LL = La Liga; Mean ± SD = mean and standard deviation; *p*‐value (*) = significant after Bonferroni correction; *R* ^2^ (marg.) = marginal *R* ^2^: explained variance of the fixed effects; *β* (95% CI) = standardised estimate and 95% confidence interval.

### 3.1 A‐League

A small effect (*β*: 0.15–0.21) existed for lower passing volume in higher T (3.4 passes; 95% CI: −5.4 to −1.4 and *p* = 0.002) and WBGT (5.8 passes; 95% CI: −5.4 to −1.4 and *p* = 0.002). This reduction was present in short passes for both T (−3.3 passes; 95% CI: −5.4 to −1.2 and *p* = 0.002) and WBGT (−5.9 passes; 95% CI: −8.4 to −3.3 and *p* < 0.001) but not long passes for neither T (−0.21 passes; 95% CI: −0.53–0.11 and *p* = 0.19) nor WBGT (−0.09 passes; 95% CI: −0.48–0.31 and *p* = 0.67). There was a small effect for lower overall touches (*β*: 0.15–0.21) in higher T (−4.3 touches; 95% CI: −6.3 to −2.4 and *p* < 0.001) and WBGT (−6.9 touches; 95% CI: −9.3 to −4.5 and *p* < 0.001), though this association was not significant for touches in the offensive third for neither T (−0.54 touches; 95% CI: −1.39 to −0.31 and *p* = 0.21) nor WBGT (−1.33 touches; 95% CI: −2.38 to −0.28 and *p* = 0.01). The number of take‐ons was also not associated with neither T (−0.17 take‐ons; 95% CI: −0.32 to −0.01 and *p* = 0.03) nor WBGT (−0.25 take‐ons; 95% CI: −0.44 to −0.06 and *p* = 0.01), but the rate of successful take‐ons was higher by 0.3% (95% CI: 0.2%–0.5% and *p* < 0.001) and 0.4% (95% CI: 0.2%–0.6% and *p* < 0.001) when T and WBGT were higher, resulting in a small effect (*β*: 0.16–0.18). The overall number of turnovers was lower by 1.3 turnovers (95% CI: −1.5 to −1.0 and *p* < 0.001) and 1.4 turnovers (95% CI: −1.8 to −1.2 and *p* < 0.001) when T and WBGT were higher, resulting in a small effect (*β*: 0.14–0.15).

### 3.2 Bundesliga 1

There were fewer short passes when T (−2.0 passes; 95% CI: −3.0 to −1.0 and *p* = 0.001) or WBGT (−2.4 passes; 95% CI: −3.6 to −1.2 and *p* = 0.001) were higher, resulting in a small effect (*β*: 0.21–0.28). No association was evident for the number of long passes for T (+0.13 passes; 95% CI: −0.24–0.50 and *p* = 0.48) or WBGT (+0.15 passes; 95% CI: −0.29–0.58 and *p* = 0.49). There was a small effect (*β*: 0.17–0.18) for fewer passes into the final third of the opponent in higher T (−0.3 passes; 95% CI: −0.4 to −0.1 and *p* = 0.001) and WBGT (−0.3 passes; 95% CI: −0.5 to −0.1 and *p* = 0.002). The volume of touches was lower by 3.2 touches (95% CI: −5.1 to −1.4 and *p* = 0.001) per degree T was higher and 4.0 touches (95% CI: −6.1 to −1.8 and *p* = 0.001) per degree WBGT was higher, resulting in a small effect (*β*: 0.20–0.26). Touches in the final third were lower in higher T (−1.1 touches; 95% CI: −1.7 to −0.5 and *p* < 0.001) or higher WBGT (−1.3 touches; 95% CI: −2.0 to −0.6 and *p* < 0.001). The overall number of turnovers was lower by 1.1 turnovers (95% CI: −1.7 to −0.6 and *p* < 0.001) with each degree higher in T or 1.3 turnovers (95% CI: −2.0 to −0.7 and *p* < 0.001) with each degree higher in WBGT, resulting in a small effect (*β*: 0.22–0.23).

### 3.3 Bundesliga 2

There were fewer short passes associated with higher T (−1.8 passes; 95% CI: −2.5 to −1.1 and *p* < 0.001) and WBGT (−2.0 passes; 95% CI: −2.7 to −1.2 and *p* < 0.001), resulting in a small effect for both T and WBGT (*β*: 0.28), but no effects on long passes was observed for T (+0.34 passes; 95% CI: 0.03–0.65 and *p* = 0.03) or WBGT (+0.40 passes; 95% CI: 0.05–0.75 and *p* = 0.02). There were fewer touches associated with higher T (−3.2 touches; 95% CI: −4.02 to −1.46 and *p* < 0.001) and WBGT (−3.4 touches; 95% CI: −4.8 to −2.1 and *p* < 0.001), touches in the offensive third with higher T (−1.4 touches; 95% CI: −2.0 to −0.9; *p* < 0.001) and WBGT (−1.6 touches; 95% CI: −2.2 to −1.0 and *p* < 0.001) as well as take‐ons with higher T (−0.2 take‐ons; 95% CI: −0.3 to −0.1 and *p* < 0.001) and WBGT (−0.2 take‐ons; 95% CI: −0.4 to −0.1 and *p* = 0.001), resulting in small to moderate effects (*β*: 0.18–0.30). The overall number of turnovers was lower by 1.3 turnovers (95% CI: −1.8 to −0.7 and *p* < 0.001) per degree T and 1.4 turnovers (95% CI: −2.1 to −0.9 and *p* < 0.001) per degree WBGT was higher, resulting in a small effect for both T and WBGT (*β*: 0.26).

### 3.4 La Liga

The total number of passes was lower by 2.2 passes (95% CI: −3.5 to −0.9; *p* < 0.001) per degree T and 2.9 passes (95% CI: −4.5 to −1.4; *p* < 0.001) per degree WBGT was higher, resulting in a small effect (*β*: 0.14–0.16). This effect was not evident in short passes for T (−0.93 passes; 95% CI: −1.83–0.04 and *p* = 0.04) or WBGT (−1.24 passes; 95% CI: −2.31 to −0.16; *p* = 0.02) but was evident for long passes for both T (−0.5 passes; 95% CI: −0.8 to −0.3 and *p* < 0.001) and WBGT (−0.8 passes; 95% CI: −1.1 to −0.4 and *p* < 0.001). The pass‐success rate was higher with higher T (0.1%; 95% CI: 0.0–0.2 and *p* < 0.001) and WBGT (0.1%; 95% CI: 0.0–0.2 and *p* = 0.001), resulting in a small effect (*β*: 0.14–0.15). There was a small effect (*β*:0.18–0.20) for less overall touches with each degree higher in T (−2.7 touches; 95% CI: −4.0 to −1.5 and *p* < 0.001) and WBGT (−3.7 touches; 95% CI: −5.2 to −2.1 and *p* < 0.001). The rate of successful take‐ons was lower with higher T (−0.4%; 95% CI: −0.5–0.2 and *p* < 0.001) and WBGT (−0.5%; 95% CI: −0.6–0.3 and *p* < 0.001). The number of turnovers was lower by 1.6 turnovers (95% CI: −2.1 to −1.1 and *p* < 0.001) per degree T and 2.0 turnovers (95% CI: −2.6 to −1.4; *p* < 0.001) per degree WBGT was higher, resulting in a small effect for both T and WBGT (*β*: 0.28).

### 3.5 Combined League Effects

**FIGURE 1:**
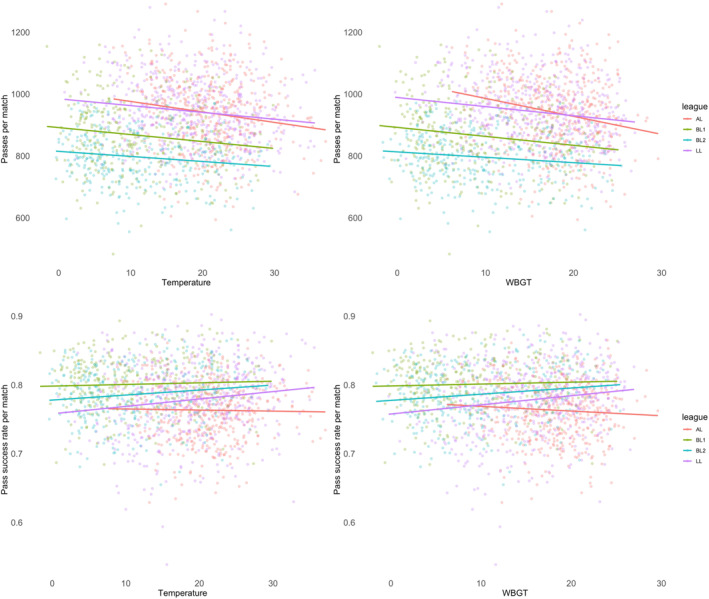
The relationship of passes and pass‐success rate with temperature and wet‐bulb globe temperature across all four leagues.

**FIGURE 2:**
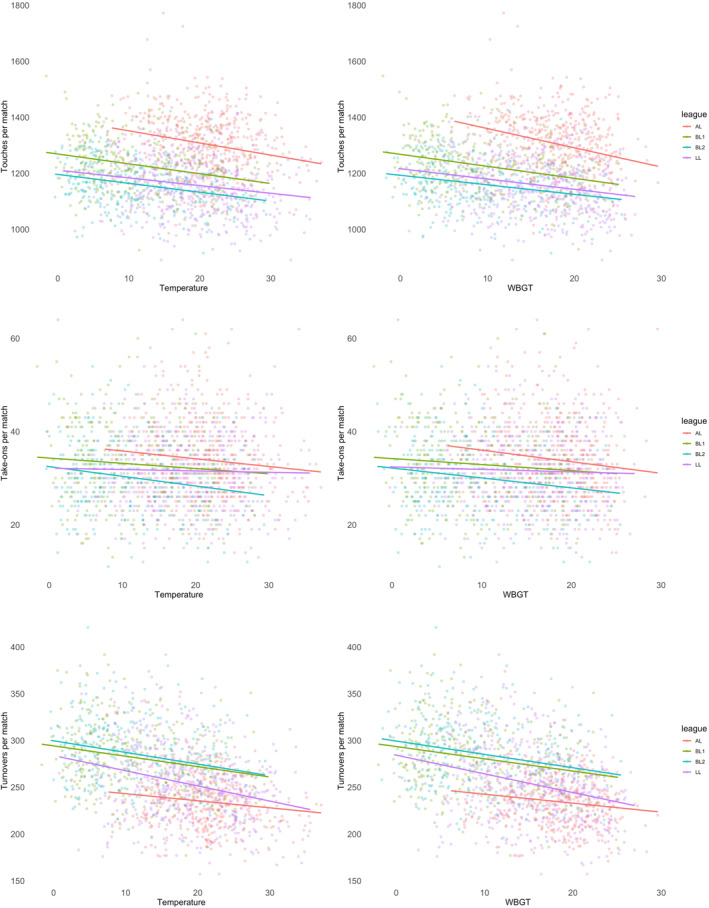
The relationship of touches, take‐ons and turnovers with temperature and wet‐bulb globe temperature across all four leagues.

When combining all data, players performed 2.3 passes (95% CI: −3.1 to −1.5 and *p* < 0.001) less per degree T and 3.1 passes (95% CI: −4.0 to −2.1 and *p* < 0.001) less per degree WBGT was higher, resulting in small effects (*β*: 0.17–0.19). This was also evident in short passes, which were lower in higher T (−1.8 passes; 95% CI: −2.4 to −1.8 and *p* < 0.001) and WBGT (−2.5 passes; 95% CI: −3.2 to −1.7 and *p* < 0.001), but not in long passes for neither T (−0.12 passes; 95% CI: −0.28–0.03 and *p* = 0.13) nor WBGT (−0.14 passes; 95% CI: −0.32–0.05 and *p* = 0.15). The rate of successful passes was slightly higher when T (0.1%; 95% CI: 0.0–0.1 and *p* = 0.002) or WBGT (0.1%; 95% CI: 0.0–0.1 and *p* = 0.001) was higher (Figure 1). The number of passes into the final third was lower in higher T (−0.2 passes; 95% CI: −0.3 to −0.1 and *p* = 0.001) or WBGT (−0.2 passes; 95% CI: −0.3 to −0.1 and *p* = 0.002), but the number of key passes resulting in a shot or goal was not associated with T (0.00 passes; 95% CI: −0.03–0.04 and *p* = 0.83) or WBGT (−0.01 passes; 95% CI: −0.05–0.03 and *p* = 0.65). There were 3.3 (95% CI: −4.1 to −2.4 and *p* < 0.001) fewer touches per degree higher in T and 4.2 (95% CI: −5.2 to −3.3 and *p* < 0.001) fewer touches per degree higher in WBGT, resulting in small effects (*β*: 0.23–0.26). Touches in the offensive third were lower in higher T (−0.9 touches; 95% CI: −1.2 to −0.6 and *p* < 0.001) or WBGT (−1.2 touches; 95% CI: −1.6 to −0.8 and *p* < 0.001) and the number of take‐ons was lower when T (−0.1 take‐ons; 95% CI: −0.2 to −0.1 and *p* < 0.001) or WBGT (−0.1 take‐ons; 95% CI: −0.2 to −0.1 and *p* < 0.001) was higher (Figure 2). Finally, there were no associations found for the number of tackles (T: −0.03 tackles; 95% CI: −0.08–0.03 and *p* = 0.30 and WBGT: −0.03 tackles; 95% CI: −0.10–0.03 and *p* = 0.32), fouls (T: −0.02 fouls; 95% CI: −0.06–0.03 and *p* = 0.45 and WBGT: −0.00 fouls; 95% CI: −0.05–0.04 and *p* = 0.90) and cards (T: +0.01 cards; 95% CI: −0.01–0.03 and *p* = 0.33 and WBGT: +0.02 cards; 95% CI: −0.00–0.04 and *p* = 0.08), but turnovers were lower for each degree T (−1.3 turnovers; 95% CI: −1.5 to −1.0 and *p* < 0.001) or WBGT (−1.5 turnovers; 95% CI: −1.8 to −1.2 and *p* < 0.001) was higher, resulting in small effects (*β*: 0.28–0.29).

## 4 Discussion

This study investigated the relationship of environmental conditions (T and WBGT) on match play PI's in four professional football leagues. Although definitions of PI's were sometimes different across the leagues (Table 1), overall trends were evident. In general, shooting PI's were not associated with changes in either T or WBGT but passing, dribbling and defending PI's were. A significantly lower number of passes, particularly short passes, and a slightly higher pass rate were associated with higher T and WBGT. Noteworthy was also a lower number of passes into the offensive final third of the pitch, although key passes ‐ leading to a shot or goal ‐ were not associated with T or WBGT. Further, the number of touches (touches in the final third), take‐ons and turnovers were also lower in higher T and WBGT. Therefore, the hypothesis is partially accepted: some PI‐volumes were significantly reduced (passes, short passes, passes into the final third, touches, touches in the final third, take‐ons and turnovers), while others were not (goals, shots, long passes, key passes, tackles and fouls cards) and performance quality metrics were maintained (take‐on success rate and pass distance) or increased (pass‐success rate). Generally, T and WBGT only described a small amount of PI variance, and the standardised estimates show only small effects.

The number of shots is an important PI associated with success in football matches (Lepschy, Wäsche, and Woll 2020; Rampinini et al. 2009). In this investigation, the number of shots and goals were not associated with either T or WBGT, which is in line with previous research (Chmura et al. 2021). Hence, although other football actions (running, passing and ball involvements) were reduced in hotter conditions, this may enable players to manage fatigue and maintain the ability to create goal‐scoring opportunities (Mohr et al. 2012; Nassis et al. 2015).

Passing has been found to be increased in matches won (Broich et al. 2014; Chmura et al. 2021), in more successful teams (Liu et al. 2015), and to be associated with scoring more goals (Bostanci et al. 2018). Our study found that the number of passes was lower in hotter T or WBGT in the AL and LL but not in BL1 and BL2. Estimates and effect sizes in BL1 and BL2 were similar, but relationships were nonsignificant after post hoc corrections. When combining the data from all four leagues, this resulted in a small effect, comparable to other research (Konefał et al. 2021). Pass‐success rates were not significantly associated with either T or WBGT in the AL, BL1 and BL2 individually but were higher in warmer conditions in the LL and when combining all match data, which were also found in previous research (Mohr et al. 2012; Nassis et al. 2015; Zhou et al. 2019). Increased pass rates may result from lower opponent pressure, possibly due to lower running distances covered at high intensities in hotter conditions, which were reduced by 4%–6% when WBGT increased by 10°C (Schwarz et al. 2024). Novel findings of this study include the reduction of short passes (evident in AL, BL1, BL2 and combined) rather than long passes (evident in LL) and the observation of a lower number of passes into the final third in higher T or WBGT. This is especially important, as a higher number of short passes has been associated to with more successful football teams (Rampinini et al. 2009) and passes into the opponent's final third have been found to be most effective in creating goal scoring opportunities (Rein, Raabe, and Memmert 2017). However, the number of key passes, leading to a shot or goal is not associated with changes in either T or WBGT. The overall findings on reduced passing volumes may depict a more static and slower match play, which is possibly a reaction to the assumed increased heat strain and confirms the findings of the only experimental study, which came to a similar conclusion, comparing a match in hot versus neutral temperatures (Mohr et al. 2012). Similar to a reduced physical performance in the heat (Draper et al. 2022; Schwarz et al. 2024), this might be part of a pacing strategy, where players reduce the overall amount of passing activity, whilst maintaining key passes, pass quality and the overall distance passes cover. Whether this occurs subconsciously, because of elevated fatigue in the heat (Nybo, Rasmussen, and Sawka 2014) or consciously in the form of prospectively applied match tactical strategies (Mohr et al. 2012) remains in question.

It has been shown that ball contacts are an important parameter for winning football matches (Broich et al. 2014) and more successful teams have more ball touches and attempt more dribbling take‐ons (Liu et al. 2015; Rampinini et al. 2009). This study showed a lower number of touches in each of the observed leagues when T or WBGT was higher. Importantly, this reduction was also evident in the offensive third in BL1, BL2 and when combining all match data. Also, the number of dribbling take‐ons was reduced in BL2 and when combining all match data. This has not been previously investigated but is in line with the observed reductions in passing PI's and further describes a lower amount of technical activity and match actions performed in hotter conditions, again possibly due to the pacing of game involvements.

Defensive qualities are an equally important factor in football performance. For example, a higher number of tackles was found to be performed by more successful teams in the Italian Serie A (Rampinini et al. 2009). Our study observed no associations between environmental conditions and the number of tackles, fouls and cards. Although speculative, the maintenance of these events may result from adjustments of other gameplay aspects in the heat, observed in this study, as these PI's might be critical in preventing goal scoring opportunities. Nevertheless, the number of turnovers was lower in warmer conditions in each of the observed leagues, which is in line with previous studies suggesting a lower opponent pressure in matches held under hot environmental conditions (Mohr et al. 2012; Nassis et al. 2015). The data of this study suggest further that this reduction in turnovers could also be linked to the lower number of attempted passes, touches and take‐ons.

Exercising in hot conditions is a concern for sporting and football organisations, which have been introducing various heat policies and guidelines, including different thresholds and indexes for their recommendations (Gouttebarge et al. 2023; Nassis et al. 2024). In this investigation, there was no difference in whether T or WBGT was used as the independent variable in the models but WBGT was estimated. A measurement of WBGT, including actual air flow and mean radiant temperature at the athletes' field of play may be necessary, albeit unlikely in research with large datasets (Racinais et al. 2023). Nevertheless, when considering the implementation of heat guidelines and policies, the use of T should not be neglected. Especially in lower competition levels, using WBGT is not feasible due to its high financial and organisational demand, which might present a barrier to implementing guidelines. Although the observed effects in this study remained small, football federations should be aware of the possible detrimental effects on football match play, which was found whilst the data contained only a small number of matches in hot environmental conditions. With an expected increase in exposure to hotter conditions in the future, these effects may become more pronounced. Therefore, environmental conditions and their associations with performance and health aspects of football players should be monitored further and federations could consider the preventive implementation of heat mitigating policies to maintain a low exposure to heat stress and consequently the quality of play (Gouttebarge et al. 2023).

Although the outcomes of this study are important to fill an existing gap in knowledge, there remain some limitations that could be addressed in future investigations. By including the leagues as random effects in the mixed models, we tried to account for differences in data sources for match characteristics and environmental conditions and aimed to detect universal effects across different geographical settings and football competitions. A more standardised line of data collection would be preferable, though the advantages of combining multiple leagues and increasing the external validity of the investigation might outweigh the limitations of a less standardised data collection. Additionally, measuring environmental conditions directly at the match venues would improve the accuracy of the heat stress observed by players (Chalmers, Anderson, and Jay 2020; Racinais et al. 2023), but the chosen retrospective approach is appropriate for investigating performance markers and enables the inclusion of a large sample of matches. Furthermore, the presented data were analysed at a level per match, disregarding the influence of each team or even individual players. Future research could aim at including individual teams and try to link observed effects to match outcomes. Further, including physical performance data alongside match‐play characteristics would describe the effect of hot conditions more holistically. Future studies should also aim to investigate these associations in a female cohort. Finally, although our data represented a wide range of environmental conditions, it can be argued that the number of observations in hot conditions is low, with only 3 matches at extreme, 10 matches at high, 142 matches at moderate and 331 matches at a low heat risk, whereas there were 1099 matches at no heat risk (Roberts et al. 2023). This might be a reason for effect sizes remaining small, as hotter conditions could potentially increase the observed effects (Schmit et al. 2015). More matches in such environments would be needed to better understand the effects of very hot conditions.

## 5 Conclusion

In conclusion, across four different football leagues, a higher T or WBGT led to alterations in match play. Mainly, small effects on lower amounts of (short) passes, touches and take‐ons were observed, depicting a more static and slower match play. Critically, this involves passes into and touches in the final third. Football teams should be aware of these associations, possibly alter match tactical approaches or consider applying heat‐mitigating strategies. Methods, such as adequate hydration, cooling before and during the half‐time of matches or performing shorter warm‐ups, have been suggested; however their effectiveness in the field is yet to be proven (Gouttebarge et al. 2023; Nassis et al. 2024). This is also true for recent investigations of in‐play cooling breaks, which have been introduced by football organisations (Brown et al. 2024; Chalmers et al. 2019). As these associations were found in a dataset containing a relatively low number of matches in hot conditions, football federations could consider this information for implementing and updating existing heat policies to prevent exposure to hotter matches, which may exaggerate the observed effects.

## Ethics Statement

The ethical approval was granted by the Ethics Committee of the Faculty for Human and Business Sciences of Saarland University (Ref No.: 23–14).

## Conflicts of Interest

The results of the study are presented clearly, honestly and without fabrication, falsification or inappropriate data manipulation. Edgar Schwarz receives a scholarship from the ‘Deutsche Fußball Liga GmbH’ (DFL) that is operating the German Bundesliga 1 and 2. Tim Meyer is the chairman of the medical committee of the German FA (Deutscher Fußball Bund, DFB) and Union of European Football Associations (UEFA) as well as head of a DFL working group entitled ‘Medicine in Professional Football’. Rob Duffield is the Head of Research and Development at Football Australia. The authors declare no further conflicts of interest.

## Data Availability

Environmental data can be accessed through Meteostat.com. Match‐play data for the Bundesliga 1, Bundesliga 2 and LaLiga are publicly available at ‘FBREF—Football Stats and History’ and can be accessed via their website https://fbref.com/en/. Data from the A‐League and Bundesliga were shared by the league organisation and cannot be shared outside the research team without their prior permission. Hence, the full dataset containing data for all four leagues is currently only available upon reasonable request to the corresponding author ES.
